# Bilastine Reimagined: A Comprehensive Exploration of Pruritus Management With a Novel Antihistamine

**DOI:** 10.7759/cureus.71232

**Published:** 2024-10-10

**Authors:** B. B. Mahajan, Pravin Banodkar, Gaurav Bhardwaj, Narendra Gokhale, K. C. Nischal, S. K. Shahirar Ahmed, Akhilesh Sharma, Mayur Mayabhate, Tejashri A Jaju

**Affiliations:** 1 Dermatology, Guru Gobind Singh (GGS) Medical College, Faridkot, IND; 2 Dermatology, Saifee Hospital, Mumbai, IND; 3 Dermatology, Skin Well Clinic, New Delhi, IND; 4 Dermatology, Sklinic, Indore, IND; 5 Dermatology, Nirmal Skin and Hair Clinic, Bengaluru, IND; 6 Dermatology, Calcutta National Medical College and Hospital, Kolkata, IND; 7 Medical Affairs, Alkem Laboratories Ltd., Mumbai, IND

**Keywords:** bilastine, chronic urticaria, histamine h1 receptor, pharmacokinetics, pruritus, wheal and flare response

## Abstract

Managing pruritic conditions is essential due to their significant impact on patients' quality of life. Chronic urticaria (CU), characterized by persistent itching and hives, severely affects daily activities and sleep. CU includes chronic inducible urticaria and chronic spontaneous urticaria, with the latter lacking identifiable triggers, making treatment especially challenging. CU is a condition that occurs across all age groups, with a higher prevalence among young adults and middle-aged women. Current antihistamine treatments include first-generation antihistamines, which are associated with sedation, and second-generation antihistamines such as cetirizine and fexofenadine, which cause less sedation but have varying efficacy and safety profiles. Bilastine, a novel second-generation H1-antihistamine, offers advantages due to its potent antihistaminic activity, rapid onset of action, and minimal sedation. This narrative review thoroughly synthesizes the evidence for the efficacy and safety of bilastine in treating CU and other pruritic conditions. By analyzing clinical trials, real-world evidence, and comparative studies, this review aims to provide a comprehensive understanding of the role of bilastine in managing pruritic conditions, emphasizing its pharmacokinetic properties, clinical efficacy, and safety profile compared to other antihistamines.

## Introduction and background

Managing pruritic conditions is crucial due to their significant impact on patients' quality of life. Pruritis, or severe itching, can result from various conditions, each with varying prevalence. Common causes include atopic dermatitis, contact dermatitis, seborrheic dermatitis, psoriasis, scabies, and systemic conditions such as thyroid, renal, or hepatic disease [1]. Among these, chronic urticaria (CU) stands out for its particularly detrimental effects on daily activities and sleep, leading to reduced quality of life and psychological distress. CU is characterized by persistent itching and hives and is further classified into chronic inducible urticaria, where symptoms are triggered by specific external factors, and chronic spontaneous urticaria (CSU), which lacks identifiable triggers, making treatment more challenging [2,3]. The absence of identifiable triggers in CSU often necessitates a multifaceted treatment approach.

The point prevalence of CU ranges from 0.5% to 1% of the global population across all age groups. It is more common in young people and middle-aged females and can persist for several years in many patients. In 25%-75% of patients, the duration exceeds one year. Unfortunately, large-scale studies in the Indian context are lacking to accurately suggest the prevalence of various CU subtypes. CSU, a self-limiting disease with a variable duration, shows that 21%-47% of patients achieve remission within one year, whereas 34%-45% attain remission within five years [4].

Current antihistamine treatments, including first-generation (e.g., diphenhydramine) and second-generation (e.g., cetirizine, fexofenadine) antihistamines, are commonly used to manage pruritic conditions. First-generation antihistamines, such as diphenhydramine, chlorpheniramine, and hydroxyzine, are often associated with sedation and cognitive impairment [5]. Second-generation antihistamines, such as cetirizine, loratadine, and fexofenadine, have a reduced ability to cross the blood-brain barrier, resulting in less sedation and cognitive impairment, though their efficacy and safety profiles may vary [6]. CSU often requires high doses of second-generation antihistamines or combination therapy to achieve adequate symptom control. The European Academy of Allergy and Clinical Immunology guidelines recommend up-dosing second-generation antihistamines up to four times the standard dose for patients with refractory CSU [7]. In this context, bilastine, a novel second-generation H1-antihistamine, offers advantages over existing treatments due to its potent antihistaminic activity, rapid onset of action, and minimal sedation [8]. Bilastine provides effective relief from symptoms of CU and other pruritic conditions without the central nervous system (CNS) side effects commonly associated with older antihistamines [9].

Bilastine has gained attention due to its favorable safety profile and efficacy in relieving symptoms of CU and other pruritic conditions. As patient responses can vary widely and there is a need for tailored treatment strategies, now is an ideal time to review its efficacy and safety comprehensively. This narrative review aims to thoroughly evaluate the efficacy and safety of bilastine in treating CU and other pruritic conditions by analyzing clinical trials, real-world evidence, and comparative studies.

## Review

Review methodology

We chose a narrative review approach to synthesize a broad range of evidence, allowing for an in-depth examination of the efficacy and safety of bilastine across different pruritic conditions. The literature search was conducted using several databases such as PubMed, MEDLINE, and Cochrane Library, using keywords “bilastine,” “chronic urticaria,” “pruritus,” “efficacy,” and “safety.” We included clinical trials, real-world studies, and meta-analyses published in English. Studies were selected based on their relevance to the efficacy and safety of bilastine in pruritic conditions. A total of 46 studies were reviewed and included in this narrative review.

Pharmacology of bilastine compared to other antihistamines

Bilastine, a selective H1-antihistamine with a high affinity for peripheral H1 receptors, effectively prevents histamine-induced symptoms such as itching and hives [10]. Extensive studies have characterized the pharmacokinetic and pharmacodynamic properties of bilastine, highlighting its advantages over other antihistamines such as cetirizine, fexofenadine, loratadine, and desloratadine. Bilastine is rapidly absorbed in the absence of food, reaching a mean peak plasma concentration of 220 ng/mL approximately one to 1.31 hours after both single and multiple dosing. A high-fat diet is known to interfere with the absorption of bilastine. The oral bioavailability of bilastine is 60.7% (90% confidence interval (CI), 53.79-67.56). The drug exhibits linear pharmacokinetics in the 2.5 to 220 mg dose range in healthy adult subjects, with no evidence of accumulation after 14 days of treatment [11-13].

The peripheral selectivity of bilastine is attributed to its limited brain uptake, mediated by its binding to P-glycoprotein which is largely due to its limited ability to cross the blood-brain barrier. This characteristic minimizes CNS penetration and reduces the risk of sedation [14]. Comparatively, other second-generation antihistamines such as cetirizine, loratadine, and fexofenadine exhibit varying degrees of peripheral selectivity. Cetirizine, for example, has a moderate affinity for H1 receptors in the brain, leading to a higher incidence of sedation compared to bilastine [15]. Loratadine and fexofenadine, like bilastine, have minimal CNS penetration, but bilastine's brain H1 receptor occupancy is among the lowest [16,17]. Unlike loratadine and desloratadine, bilastine undergoes minimal hepatic metabolism and is excreted largely unchanged (96% of the administered dose) within 24 hours through the urine and feces [11]. The half-life of bilastine is approximately 14.5 hours, with a clearance rate of 18.1 L/h [8]. In comparison (1) Cetirizine has a bioavailability of 70%, a volume of distribution (Vd) of 0.5 L/kg, and a half-life of 8.3 hours, with significant protein binding at 93% [15,18]. (2) Fexofenadine has a lower bioavailability of 30%-41%, a Vd of 5.4-5.8 L/kg, and a half-life of 14.4 hours, with food significantly reducing its bioavailability [19,20]. (3) Loratadine has a bioavailability of 40% and a Vd of 119 L. It is extensively metabolized in the liver, leading to a longer half-life of 27 hours for its active metabolite, desloratadine. (4) Desloratadine has a bioavailability of 50%, a Vd of 49.2 L, and a half-life of 27 hours [21]. The H1 receptor occupancy in the brain varies among second-generation H1 antihistamines. Bilastine and fexofenadine exhibit no detectable brain penetration, minimizing CNS side effects, whereas levocetirizine, loratadine, and cetirizine show moderate to high brain penetration, leading to higher receptor occupancy.

While the pharmacokinetic data in the Indian context are limited and warrant further exploration, a Japanese study revealed that bilastine exhibits similar pharmacokinetic and pharmacodynamic characteristics in healthy Japanese patients compared with Caucasian patients [22]. Bilastine is quickly absorbed and exhibits consistent pharmacokinetics across various doses, ensuring stable therapeutic levels without significant accumulation. Its minimal metabolism and primarily unchanged excretion also reduce the potential for drug-drug interactions, offering a safer profile for patients on multiple medications [23]. The clinical profile differences between various second-generation H1 antihistamines are provided in Table 1 [15,24-34].

**Table 1:** Clinical profile differences between various second-generation H1 antihistamines *Information was also sourced from the Summary of Product Characteristics for each individual compound (accessible at http://www.medicines.org.uk/emc/).

Parameter	Bilastine	Cetirizine	Levocetirizine	Loratadine	Fexofenadine
Histamine H1-receptor selectivity	Ki 44.15 ± 6.08 nM	Ki 143.12 ± 16.35 nM			Ki 246 ± 40.7 nM
H1 receptor occupancy in brain (%)	–3.92%	26.0%	8.1%	11.7%	−8.0%
t_max_ (hours)	1.3	1.0	0.9	1.0-1.5	1-3
t_1/2_ (hours)	14.5	10.0	7.9	8.4	11-15
C_max_ (ng/ml)	220	257	203	4.5 (ng/mL)	427
V_d_ (volume of distribution)	1.29 L/kg	0.56 L/kg	0.3 L/kg	119 L	5.4 L/kg
Protein binding	~84%	93%	95%	97%	60-70%
Metabolism	Minimal	Limited (CYP3A4, 2D6)	Limited (CYP3A4, 2D6)	Extensive (CYP3A4)	Minimal (CYP3A4)
Excretion	Renal (95%)	Renal (60-70%)	Renal (85%)	Renal and fecal	Fecal (80%)
Elimination pathways	Renal	Renal	Renal	Hepatic (CYP3A4)	Biliary excretion
Citation	^[24–26]^	^[15,24,27]^	^[28–31]^	^[32–34]^	^[24,31]^

In pediatric populations, bilastine has shown pharmacokinetic properties comparable to those observed in adults. A 10-mg daily dose provides similar plasma drug concentrations and pharmacodynamic effects in children as the 20-mg dose in adults. In children aged two to <12 years, the area under the curve (AUC)0-24 and Cmax values are similar to those in adults, with pediatric/adult ratios for AUC0-24 and Cmax close to unity (0.93 and 0.91, respectively). These findings support the appropriateness of the 10-mg pediatric dose of bilastine, which has a safety profile comparable to placebo [35-37]. PK/PD simulations have further validated the use of a 10-mg daily dose in children aged two to <12 years, ensuring effective management of pruritic conditions without the need for dose adjustments [36,38].

The versatility of bilastine is also highlighted by its formulation in oro-dispersible tablets and oral solutions, which are bioequivalent to the conventional tablet formulation. The oro-dispersible tablet shows bioequivalence even when administered without water, showcasing the suitability of bilastine for pediatric use and enhancing medication palatability [39]. The minimal metabolism of bilastine, reduced risk of sedation due to limited blood-brain barrier penetration, rapid onset of action, consistent effectiveness regardless of food intake, and once-daily dosing position it as a preferred option for the treatment of CU and other pruritic conditions.

Clinical efficacy of bilastine compared to cetirizine/levocetirizine

A comparison of the clinical efficacy and safety of bilastine with cetirizine, levocetirizine, and fexofenadine is provided in Table 2.

**Table 2:** Comparison of clinical efficacy and safety of bilastine with cetirizine, levocetirizine, and fexofenadine CSU - chronic spontaneous urticaria, MTSS - modified total symptom score, CU - chronic urticaria, VAS - visual analog scale, UAS7 - urticaria activity score, TSS - total symptom score

Study	Design and population	Drugs compared	Key findings	Conclusion
Sinha et al. (2023) [40]	70 patients with CSU, randomized, 6-week treatment	bilastine 20 mg vs. cetirizine 10 mg	Bilastine showed a greater reduction in MTSS (p<0.05) and lower VAS for sedation. Significant reduction in monocytes (p=0.042) and basophils (p≤0.0001) in the bilastine group.	Bilastine is more effective with lower sedation compared to cetirizine.
Shah et al. (2022) [41]	110 patients with CSU unresponsive to standard doses, randomized	bilastine vs. fexofenadine vs. levocetirizine	Bilastine significantly improved UAS7 (p<0.05) and quality of life (CU-Q2oL, p<0.05) compared to levocetirizine, with lower somnolence and adverse events.	Bilastine offers superior efficacy and safety in CSU compared to levocetirizine, and better quality of life than fexofenadine.
Chen et al. (n.d.) [42]	Chinese patients with CSU, randomized, double-blind, 4-week treatment	bilastine 20 mg vs. levocetirizine 5 mg	Bilastine showed non-inferiority in TSS reduction, with comparable DLQI scores by day 28.	Bilastine is as effective as levocetirizine with similar quality of life improvements.
Podder et al. (2020) [43]	Patients with moderate-to-severe CSU, randomized, 4-week treatment	bilastine 20 mg vs. levocetirizine 5 mg	Bilastine significantly reduced UAS7 scores (p=0.03) and DLQI scores (p<0.001) with lower sedation (p=0.04).	Bilastine is more effective in reducing urticaria symptoms and improving quality of life with less sedation compared to levocetirizine.
Zuberbier et al. (2010) [44]	Patients with moderate-to-severe CU, randomized, placebo-controlled	bilastine 20 mg vs. levocetirizine 5 mg vs. placebo	Bilastine significantly reduced TSS, DLQI, and sleep disruption (p<0.001 for all) compared to placebo, with similar efficacy and safety as levocetirizine.	Bilastine and levocetirizine are equally efficacious and well-tolerated, with bilastine offering additional benefits over placebo.
Shah et al. (2022) [41]	Patients with CU, randomized, double-blind	bilastine vs. fexofenadine	Bilastine significantly improved CU-Q2oL scores (p<0.05) and was associated with lower sedation (p<0.05) compared to fexofenadine.	Bilastine provides better quality of life and lower sedation in CU patients compared to fexofenadine.

Few studies in the Indian population have compared the safety and efficacy of bilastine with other second-generation antihistamines such as cetirizine. In a study involving 70 patients with CSU, participants received either cetirizine 10 mg or bilastine 20 mg once daily for six weeks. The mean reduction in the modified total symptom score (MTSS) was significantly greater (p<0.05) in the bilastine group compared to the cetirizine group. Additionally, cetirizine was associated with a significant increase in the visual analog scale (VAS) score for sedation. When comparing the mean difference in monocyte and basophil counts from baseline to six weeks, bilastine showed a significant reduction in both monocytes (p=0.042) and basophils (p≤0.0001) [40]. Another comparative study assessed the efficacy and safety of different antihistamine regimens in patients with CSU unresponsive to standard doses. This study randomized 110 patients into three groups receiving either bilastine, fexofenadine, or levocetirizine. Bilastine not only improved CSU symptoms but also showed a significant difference in urticaria activity score (UAS7) improvement at week 4 compared to levocetirizine (p<0.05). The quality of life, assessed using the CU-Q2oL, improved significantly more with bilastine compared to levocetirizine (p<0.05). Moreover, bilastine demonstrated lower somnolence and adverse events, with a statistically significant improvement in urticaria symptoms and overall patient satisfaction at the end of four weeks [41].

In a study evaluating the efficacy and safety of bilastine compared to levocetirizine in Chinese patients, the mean changes from baseline in total symptom score (TSS) were -2.54±2.59 for bilastine and -3.06±2.47 for levocetirizine, with least squares mean difference of 0.17, demonstrating non-inferiority. Investigator-assessed TSS also showed comparable decreases in both groups by day 28. The Dermatology Life Quality Index (DLQI) scores on day 28 were similar, indicating comparable quality-of-life improvements [42]. A study comparing bilastine 20 mg and levocetirizine 5 mg in moderate-to-severe CSU found a significant reduction in UAS7 scores, with bilastine being more effective (p=0.03). By the end of the treatment, 80.6% of patients in the bilastine group had their urticaria “well-controlled” (UAS≤6) compared to 51.9% in the levocetirizine group (p=0.04). Sedation was significantly less with bilastine (p=0.04). Bilastine also significantly reduced the mean DLQI score from 17.8±2.9 at baseline to 5.7±3.5 at the end of treatment, with both changes being statistically significant (p<0.001) [43]. In a comparative study of bilastine 20 mg, levocetirizine 5 mg, and placebo in CU patients with moderate-to-severe symptoms, bilastine reduced mean reflective and instantaneous TSS from baseline to a significantly greater degree than placebo (p<0.001). The DLQI, general discomfort, and sleep disruption also improved significantly in bilastine-treated patients compared to placebo-treated patients (p<0.001 for all parameters). Both bilastine and levocetirizine were equally efficacious, safe, and well-tolerated compared to placebo [44].

Overall, bilastine demonstrates superior clinical efficacy and safety compared to cetirizine and levocetirizine in treating CSU. Bilastine consistently shows greater improvements in symptom scores, lower sedation levels, and enhanced quality of life, making it a preferable option in managing this condition.

Clinical efficacy of bilastine compared to fexofenadine

Another study evaluating bilastine revealed a significant (p<0.05) improvement in quality of life assessed by CU-Q2oL compared to fexofenadine. Bilastine is well accepted as a non-sedating antihistamine as evidenced by a significant (p<0.05) VAS score between bilastine and fexofenadine [41].

Clinical efficacy of bilastine in refractory patients

Real-world data on the efficacy of bilastine in CSU is gradually accumulating. A retrospective study involving 177 CSU patients refractory to other antihistamines at double doses in India reported that 42% of patients responded to a double dose of bilastine (40 mg/day) with significant improvement in UCT scores and minimal sedation. The study demonstrated a significant increase in UCT scores from 5.0±2.2 at baseline to 8.08±5.41 at two weeks, indicating a 62% improvement. These findings underscore bilastine's effectiveness and tolerability in managing CSU symptoms refractory to standard antihistamine therapy [45]. In another real-world study conducted in India, bilastine at a licensed dose (20 mg once daily) was evaluated in patients refractory to levocetirizine at standard doses (UCT score <12). Bilastine showed notable effectiveness, with 73.48% of patients responding positively within four weeks. The mean UCT score improved from 9±2.62 at baseline to 13.05±2.78 after four weeks of treatment (p<0.05), along with improvements in VAS scores. These findings suggest that bilastine is a superior alternative to other H1-antihistamines, particularly for patients not achieving adequate symptom control with standard doses of drugs such as levocetirizine [46].

Effect of bilastine on Wheal and flare response in patients with pruritic conditions

Bilastine demonstrates significant advantages over other antihistamines in managing wheal and flare responses in patients with pruritic conditions. Studies indicate that a 20-mg dose of bilastine significantly inhibits the wheal and flare response more effectively and quickly compared to desloratadine 5 mg and rupatadine 10 mg, and it significantly reduces itching compared to placebo [47]. Both bilastine 20 mg and cetirizine 10 mg are effective in reducing histamine-induced wheal and flare responses, with bilastine showing a more rapid onset of action [48]. Moreover, bilastine 20 mg shows minimal H1-receptor occupancy, does not cause subjective sedation, and does not impair psychomotor performance, confirming its non-sedating properties [26]. It maintains its effectiveness without tolerance development, and unlike hydroxyzine, bilastine 20 mg does not induce psychomotor impairment or subjective sedation, clearly separating peripheral antihistamine effects from CNS side effects, which only appear at higher doses [49]. In summary, bilastine 20 mg offers superior inhibition of wheal and flare responses compared to desloratadine and rupatadine, with a faster onset of action and a significant reduction in itching. It matches cetirizine in effectiveness but provides a quicker onset and avoids sedation or psychomotor impairment, maintaining efficacy without tolerance development and separating peripheral effects from CNS side effects at therapeutic doses.

Comparative safety profile of bilastine with other antihistamines

Bilastine is demonstrated to be more favorable compared to other antihistamines such as cetirizine, fexofenadine, and levocetirizine, primarily due to its lower incidence of adverse events, particularly sedation (Table 3).

**Table 3:** Comparative safety profile of bilastine with cetirizine, fexofenadine, and levocetirizine CSU - chronic spontaneous urticaria, CU - chronic urticaria

Study	Design & population	Drugs compared	Key safety findings	Conclusion
Sinha et al. (2023) [40]	patients with CSU, randomized, 6-week treatment	bilastine 20 mg vs. cetirizine 10 mg	Bilastine group reported 4 adverse events compared to 13 in the cetirizine group, with significantly lower sedation rates in the bilastine group.	Bilastine has a more favorable safety profile with fewer adverse events and less sedation than cetirizine.
Podder et al. (2020) [43]	patients with moderate-to-severe CSU, randomized, 4-week treatment	bilastine 20 mg vs. levocetirizine 5 mg	Bilastine had a lower incidence of somnolence (12.9% vs. 63%, p=0.002) and fewer moderate treatment-emergent adverse events (3.6% vs. 2.1%).	Bilastine is associated with lower rates of somnolence and fewer moderate adverse events than levocetirizine.
Chen et al. (n.d.) [42]	Chinese patients with CSU, randomized, double-blind, 4-week treatment	bilastine 20 mg vs. levocetirizine 5 mg	bilastine showed a lower rate of adverse events compared to levocetirizine, particularly in terms of somnolence and headache.	Bilastine presents a safer option with fewer adverse effects compared to levocetirizine.
Shah et al. (2021) [46]	Patients with CU, randomized, double-blind	bilastine vs. levocetirizine	Bilastine had fewer overall adverse events, mostly mild and not leading to treatment discontinuation. A statistically significant reduction in adverse events was observed (p<0.05).	Bilastine is better tolerated with fewer and milder adverse events than levocetirizine.
Shah et al. (2022) [41]	Patients with CU, randomized, double-blind	bilastine vs. levocetirizine + hydroxyzine	Bilastine showed significantly fewer adverse events (p<0.05) compared to the levocetirizine-hydroxyzine combination, highlighting its superior safety profile.	Bilastine has a significantly better safety profile compared to the levocetirizine-hydroxyzine combination.
Zuberbier et al. (2010) [44]	Patients with moderate-to-severe CU, randomized, placebo-controlled	bilastine 20 mg vs. levocetirizine 5 mg vs. placebo	No serious adverse events reported for bilastine. No significant changes in laboratory tests, ECGs, heart rate, or blood pressure were observed across treatment groups.	Bilastine is well-tolerated with no serious adverse events, making it a safe alternative to levocetirizine.

Studies have shown that cetirizine had a significantly higher rate of adverse events, especially sedation, with 13 patients experiencing adverse events compared to only four patients in the bilastine group [40]. Levocetirizine was associated with a higher rate of somnolence (63% compared to 12.9% for bilastine, p=0.002) and other adverse events such as headache and fatigue. Additionally, moderate treatment-emergent adverse events were observed at a slightly lower rate in bilastine (3.6%) compared to levocetirizine (2.1%) [42,43].

Furthermore, in terms of overall adverse events, bilastine was associated with fewer events, and these were mostly mild and did not lead to treatment discontinuation [46]. In comparison to levocetirizine, bilastine showed a statistically significant reduction in adverse events (p<0.05), which may be attributed to the addition of hydroxyzine in the levocetirizine arm [41]. Moreover, bilastine had no major adverse events reported and was well-tolerated with no serious adverse events, and no significant changes in laboratory tests, electrocardiograms (ECGs), heart rate, or blood pressure across treatment groups [44,50].

Drug-drug interactions with bilastine

Understanding the pharmacokinetic interactions of bilastine with food and other substances is crucial for optimizing its therapeutic benefits. The interaction between bilastine with food does not significantly impact its peripheral antihistaminic efficacy. While there is a slight delay in the onset of action on the first day of treatment, the overall clinical efficacy of bilastine remains unaffected when co-administered with food [51]. When administered on an empty stomach, bilastine is rapidly absorbed. However, food intake slows its absorption, reducing oral bioavailability by approximately 30% with high-fat meals and 25% with low-fat meals. Therefore, it is recommended to take bilastine at least one hour before or two hours after eating. Bilastine undergoes minimal metabolism and does not interact with cytochrome P450 enzymes, making it a relatively safe option with fewer drug interactions. However, grapefruit juice significantly decreases the exposure of bilastine, reducing Cmax and AUC by 33% and 24%, respectively. This effect is likely due to the downregulation of OATP1A2 transport activity and P-glycoprotein. Additionally, bilastine is actively transported by MDR1 but is not a substrate for OCT2, OAT1, OAT3, or BCRP. Studies on the interaction between bilastine and alcohol have shown that therapeutic doses of bilastine combined with alcohol do not exacerbate psychomotor impairment beyond that caused by alcohol alone. However, higher doses (80 mg) result in more significant impairment compared to a placebo [52].

Current treatment guidelines and the role of bilastine for pruritic conditions

Managing pruritic conditions, such as CU, follows a structured approach as outlined by several clinical guidelines (Table 4). The European Academy of Allergy and Clinical Immunology, the Global Allergy and Asthma European Network, the European Dermatology Forum, and the World Allergy Organization offer well-regarded recommendations for treating urticaria. These guidelines suggest starting with non-sedating second-generation H1-antihistamines at standard doses. If patients do not experience enough relief, the dosage can be increased up to four times the usual amount. When even high doses of antihistamines are not effective, other treatments such as leukotriene receptor antagonists, omalizumab, or cyclosporine might be added to the regimen [7]. Bilastine, being a modern second-generation antihistamine, is suggested as a first-line treatment due to its potent antihistaminic activity, minimal sedative effects, and favorable safety profile. Studies have shown that bilastine is effective in reducing symptoms of CSU, providing significant relief from pruritus, and improving the quality of life for patients. Compared to other antihistamines, bilastine demonstrates a comparable or superior efficacy with a lower incidence of adverse effects. Its quick onset of action and long-lasting effect make it a suitable choice for managing pruritic conditions. Additionally, a lack of significant drug interactions and its minimal impact on cognitive and motor functions further cement its place as a valuable treatment option [40]. The recommendations synthesized from this narrative review are detailed in Table 4.

**Table 4:** Review recommendations CSU - chronic spontaneous urticaria, CU - chronic urticaria

Recommendations
Consider bilastine as a preferred first-line treatment for CU and other pruritic conditions due to its rapid onset of action and minimal sedative effects compared to older antihistamines.
Utilize bilastine, particularly at standard doses of 20 mg daily, for its demonstrated superior efficacy over other second-generation antihistamines such as cetirizine and levocetirizine in managing CU, ensuring effective symptom relief without compromising safety.
Explore higher doses (up to 40 mg/day) of bilastine for refractory CU cases, acknowledging its ability to improve symptom control while maintaining a favorable safety profile over extended use period.
For patients with refractory CSU, it is recommended to increase the dose of second-generation antihistamines up to four times the standard dose. This up-dosing strategy has been shown to improve symptom control in patients who do not respond adequately to the standard dose.
For pediatric patients aged 2–12 years, confidently prescribe bilastine at a safe and effective dose of 10 mg daily, aligning with its proven efficacy and tolerability in younger age groups.
Bilastine shows minimal impact on psychomotor performance and suitability for use in scenarios requiring attention and alertness, such as driving, even when combined with alcohol.

## Conclusions

Bilastine is a reliable and safe treatment for CU and other pruritic conditions, providing quick symptom relief with minimal sedation. The efficacy and safety profile make bilastine a valuable option among current antihistamines. Healthcare professionals should consider bilastine as a first-line treatment, particularly for patients who experience sedation with other antihistamines. While the review highlights the short-term efficacy and safety of bilastine, there is a lack of long-term studies evaluating its sustained effectiveness, particularly in chronic pruritic conditions like CU which could limit conclusions on its prolonged use. Much of the data comes from controlled clinical trials, which may not fully reflect the real-world diversity of patients, including those with comorbidities or those using multiple medications. Hence, future research should aim at longitudinal studies and real-world evidence to further confirm the efficacy and safety of bilastine. Additionally, exploring the potential of bilastine in personalized medicine and identifying relevant biomarkers could enhance its clinical application.

## References

[1] Chung BY, Um JY, Kim JC, Kang SY, Park CW, Kim HO (2020). Pathophysiology and treatment of pruritus in elderly. Int J Mol Sci.

[2] Chang J, Cattelan L, Ben-Shoshan M, Le M, Netchiporouk E (2021). Management of pediatric chronic spontaneous urticaria: a review of current evidence and guidelines. J Asthma Allergy.

[3] Papapostolou N, Xepapadaki P, Katoulis A, Makris M (2022). Comorbidities of chronic urticaria: a glimpse into a complex relationship. Front Allergy.

[4] Godse K, Patil A, De A (2022). Diagnosis and management of urticaria in Indian settings: skin allergy Research Society’s guideline-2022. Indian J Dermatol.

[5] Church MK, Maurer M, Simons FE (2010). Risk of first-generation H(1)-antihistamines: a GA(2)LEN position paper. Allergy.

[6] Xiang YK, Fok JS, Podder I, Yücel MB, Özkoca D, Thomsen SF, Kocatürk E (2024). An update on the use of antihistamines in managing chronic urticaria. Expert Opin Pharmacother.

[7] Zuberbier T, Aberer W, Asero R (2018). The EAACI/GA²LEN/EDF/WAO guideline for the definition, classification, diagnosis and management of urticaria. Allergy.

[8] Ridolo E, Montagni M, Bonzano L, Incorvaia C, Canonica GW (2015). Bilastine: new insight into antihistamine treatment. Clin Mol Allergy.

[9] Church MK, Tiongco-Recto M, Ridolo E, Novák Z (2020). Bilastine: a lifetime companion for the treatment of allergies. Curr Med Res Opin.

[10] Singh Randhawa A, Mohd Noor N, Md Daud MK, Abdullah B (2022). Efficacy and safety of bilastine in the treatment of allergic rhinitis: a systematic review and meta-analysis. Front Pharmacol.

[11] Jáuregui I, García-Lirio E, Soriano AM, Gamboa PM, Antépara I (2012). An overview of the novel H1-antihistamine bilastine in allergic rhinitis and urticaria. Expert Rev Clin Immunol.

[12] Sádaba B, Gómez-Guiu A, Azanza JR, Ortega I, Valiente R (2013). Oral availability of bilastine. Clin Drug Investig.

[13] Jauregizar N, De La Fuente L, Lucero ML, Sologuren A, Leal N, Rodríguez M (2009). Pharmacokinetic-Pharmacodynamic modelling of the antihistaminic (H1) effect of bilastine. Clin Pharmacokinet.

[14] Montoro J, Mullol J, Dávila I (2011). Bilastine and the central nervous system. J Investig Allergol Clin Immunol.

[15] Naqvi A, Patel P, Gerriets V (2024). Cetirizine. https://www.ncbi.nlm.nih.gov/books/NBK549776/.

[16] Nakamura T, Hiraoka K, Harada R (2019). Brain histamine H(1) receptor occupancy after oral administration of desloratadine and loratadine. Pharmacol Res Perspect.

[17] Simons FE, Simons KJ (2008). H1 antihistamines: current status and future directions. World Allergy Organ J.

[18] Chen C (2008). Physicochemical, pharmacological and pharmacokinetic properties of the zwitterionic antihistamines cetirizine and levocetirizine. Curr Med Chem.

[19] Craun KL, Patel P, Schury MP (2024). Fexofenadine. http://www.ncbi.nlm.nih.gov/books/NBK556104/.

[20] Lappin G, Shishikura Y, Jochemsen R (2010). Pharmacokinetics of fexofenadine: evaluation of a microdose and assessment of absolute oral bioavailability. Eur J Pharmaceut Sci.

[21] Affrime M, Gupta S, Banfield C, Cohen A (2002). A pharmacokinetic profile of desloratadine in healthy adults, including elderly. Clin Pharmacokinet.

[22] Togawa M, Yamaya H, Rodríguez M, Nagashima H (2016). Pharmacokinetics, pharmacodynamics and population pharmacokinetic/pharmacodynamic modelling of bilastine, a second-generation antihistamine, in healthy Japanese subjects. Clin Drug Investig.

[23] Jáuregui I, Ferrer M, Bartra J (2013). Bilastine for the treatment of urticaria. Expert Opin Pharmacother.

[24] Jáuregui I, Ramaekers JG, Yanai K, Farré M, Redondo E, Valiente R, Labeaga L (2016). Bilastine: a new antihistamine with an optimal benefit-to-risk ratio for safety during driving. Expert Opin Drug Saf.

[25] Sadaba B, Azanza JR, Gomez-Guiu A, Rodil R (2013). Critical appraisal of bilastine for the treatment of allergic rhinoconjunctivitis and urticaria. Ther Clin Risk Manag.

[26] Farré M, Pérez-Mañá C, Papaseit E (2014). Bilastine vs. hydroxyzine: occupation of brain histamine H1-receptors evaluated by positron emission tomography in healthy volunteers. Br J Clin Pharmacol.

[27] Tashiro M, Sakurada Y, Iwabuchi K (2004). Central effects of fexofenadine and cetirizine: measurement of psychomotor performance, subjective sleepiness, and brain histamine H1-receptor occupancy using 11C-doxepin positron emission tomography. J Clin Pharmacol.

[28] Ino H, Hara K, Honma G, Doi Y, Fukase H (2014). Comparison of levocetirizine pharmacokinetics after single doses of levocetirizine oral solution and cetirizine dry syrup in healthy Japanese male subjects. J Drug Assess.

[29] Benedetti MS, Plisnier M, Kaise J, Maier L, Baltes E, Arendt C, McCracken N (2001). Absorption, distribution, metabolism and excretion of [14C]levocetirizine, the R enantiomer of cetirizine, in healthy volunteers. Eur J Clin Pharmacol.

[30] Ino H, Shiramoto M, Eto T (2020). Levocetirizine oral disintegrating tablet: a randomized open-label crossover bioequivalence study in healthy Japanese volunteers. Clin Pharmacol Drug Dev.

[31] Hiraoka K, Tashiro M, Grobosch T (2024). Brain histamine H1 receptor occupancy measured by PET after oral administration of levocetirizine, a non-sedating antihistamine. Expert Opin Drug Saf.

[32] Sidhu G, Akhondi H (2024). Loratadine. http://www.ncbi.nlm.nih.gov/books/NBK542278/.

[33] Tenn MW, Steacy LM, Ng CC, Ellis AK (2018). Onset of action for loratadine tablets for the symptomatic control of seasonal allergic rhinitis in adults challenged with ragweed pollen in the Environmental Exposure Unit: a post hoc analysis of total symptom score. Allergy Asthma Clin Immunol.

[34] Kubo N, Senda M, Ohsumi Y (2011). Brain histamine H1 receptor occupancy of loratadine measured by positron emission topography: comparison of H1 receptor occupancy and proportional impairment ratio. Hum Psychopharmacol.

[35] Rodríguez M, Vozmediano V, García-Bea A, Novák Z, Yáñez A, Campo C, Labeaga L (2020). Pharmacokinetics and safety of bilastine in children aged 6 to 11 years with allergic rhinoconjunctivitis or chronic urticaria. Eur J Pediatr.

[36] Vozmediano V, Lukas JC, Encinas E (2019). Model-informed pediatric development applied to bilastine: analysis of the clinical PK data and confirmation of the dose selected for the target population. Eur J Pharm Sci.

[37] Majorek-Olechowska B, Slomskis T, Zollerová L, Martín I, Sánchez C, Gilaberte I, Arranz P (2024). Pharmacokinetics and safety of bilastine 10 mg/d in children aged 2 to 5 years with allergic rhinoconjunctivitis or urticaria: a phase 3 clinical trial [PREPRINT]. J Investig Allergol Clin Immunol.

[38] Vozmediano V, Sologuren A, Lukas JC, Leal N, Rodriguez M (2017). Model informed pediatric development applied to bilastine: ontogenic PK model development, dose selection for first time in children and PK study design. Pharm Res.

[39] Sádaba B, Azanza JR, García-Bea A, Labeaga L, Campo C, Valiente R (2020). Bioequivalence evaluation of three pediatric oral formulations of bilastine in healthy subjects: results from a randomized, open label, crossover study. Eur J Drug Metab Pharmacokinet.

[40] Sinha VV, Kalikar MV, Mukhi JI, Giradkar AB, Sontakke S (2023). Comparative study of efficacy and safety of cetirizine and bilastine in patients of chronic spontaneous urticaria: open-label, randomized, parallel-group study. Perspect Clin Res.

[41] Shah B, Dhoot D, Choudhary A (2022). A comparative, three-arm, randomized clinical trial to evaluate the effectiveness and tolerability of bilastine vs fexofenadine vs levocetirizine at the standard dose and bilastine vs fexofenadine at higher than the standard dose (up-dosing) vs Levocetirizine and hydroxyzine (in combination) in patients with chronic spontaneous urticaria. Clin Cosmet Investig Dermatol.

[42] Chen X, Han X, Cheng B (2024). Efficacy and safety of bilastine vs. levocetirizine for the treatment of chronic idiopathic urticaria: a multicenter, double-blind, double-dummy, phase III, non-inferiority, randomized clinical trial. Chin Med J (Engl).

[43] Podder I, Das A, Ghosh S, Biswas D, Sengupta S, Chowdhury SN (2020). Effectiveness, safety, and tolerability of bilastine 20 mg vs levocetirizine 5 mg for the treatment of chronic spontaneous urticaria: a double-blind, parallel group, randomized controlled trial. Dermatol Ther.

[44] Zuberbier T, Oanta A, Bogacka E (2010). Comparison of the efficacy and safety of bilastine 20 mg vs levocetirizine 5 mg for the treatment of chronic idiopathic urticaria: a multi-centre, double-blind, randomized, placebo-controlled study. Allergy.

[45] De A, Shah B, Banodkar PD, Dhoot D, Chitnis K, Barkate H (2023). Real-world Indian experience of switchover to bilastine 40 mg/day in CSU patient refractory to other antihistamines at double dose. Indian J Dermatol.

[46] Shah B, De A, Sarda A, Kochhar AM, Dhoot D, Deshmukh G, Barkate H (2021). Effect of bilastine on chronic spontaneous urticaria refractory to levocetrizine: real world experience in India. Dermatol Ther.

[47] Antonijoan R, Coimbra J, García-Gea C (2017). Comparative efficacy of bilastine, desloratadine and rupatadine in the suppression of wheal and flare response induced by intradermal histamine in healthy volunteers. Current Med Res Opin.

[48] Church MK (2011). Comparative inhibition by bilastine and cetirizine of histamine-induced wheal and flare responses in humans. Inflamm Res.

[49] García-Gea C, Martínez-Colomer J, Antonijoan RM, Valiente R, Barbanoj MJ (2008). Comparison of peripheral and central effects of single and repeated oral dose administrations of bilastine, a new H1 antihistamine: a dose-range study in healthy volunteers with hydroxyzine and placebo as control treatments. J Clin Psychopharmacol.

[50] Hide M, Yagami A, Togawa M, Saito A, Furue M (2017). Efficacy and safety of bilastine in Japanese patients with chronic spontaneous urticaria: a multicenter, randomized, double-blind, placebo-controlled, parallel-group phase II/III study. Allergol Int.

[51] Coimbra J, Puntes M, Gich I (2022). Lack of clinical relevance of bilastine-food interaction in healthy volunteers: a wheal and flare study. Int Arch Allergy Immunol.

[52] Paśko P, Rodacki T, Domagała-Rodacka R, Palimonka K, Marcinkowska M, Owczarek D (2017). Second generation H1 - antihistamines interaction with food and alcohol-a systematic review. Biomed Pharmacother.

